# Cost-Effectiveness of Community-Based Tobacco Dependence Treatment Interventions: Initial Findings of a Systematic Review

**DOI:** 10.5888/pcd16.190232

**Published:** 2019-12-12

**Authors:** Sarah A. Reisinger, Sahar Kamel, Eric Seiber, Elizabeth G. Klein, Electra D. Paskett, Mary Ellen Wewers

**Affiliations:** 1 Division of Health Behavior and Health Promotion, College of Public Health, Ohio State University, Columbus, Ohio; 2 Comprehensive Cancer Center, Ohio State University, Columbus, Ohio; 3 Division of Health Services Management and Policy, College of Public Health, Ohio State University, Columbus, Ohio; 4 Division of Cancer Prevention and Control, College of Medicine, Ohio State University, Columbus, Ohio; 5 Division of Epidemiology, College of Public Health, Ohio State University, Columbus, Ohio

## Abstract

**Introduction:**

Scientific literature evaluating the cost-effectiveness of tobacco dependence treatment programs delivered in community-based settings is scant, which limits evidence-based tobacco control decisions. The aim of this review was to systematically assess the cost-effectiveness and quality of the economic evaluations of community-based tobacco dependence treatment interventions conducted as randomized controlled trials in the United States.

**Methods:**

We searched 8 electronic databases and gray literature from their beginning to February 2018. Inclusion criteria were economic evaluations of community-based tobacco dependence treatments conducted as randomized controlled trials in the United States. Two independent researchers extracted data on study design and outcomes. Study quality was assessed by using Drummond and Jefferson’s economic evaluations checklist. Nine of 3,840 publications were eligible for inclusion. Heterogeneity precluded formal meta-analyses. We synthesized a qualitative narrative of outcomes.

**Results:**

All 9 studies used cost-effectiveness analysis and a payer/provider/program perspective, but several study components, such as abstinence measures, were heterogeneous. Study participants were predominantly English speaking, middle aged, white, motivated to quit, and highly nicotine dependent. Overall, the economic evaluations met most of Drummond and Jefferson’s recommendations; however, some studies provided limited details. All studies had a cost per quit at or below $2,040 or an incremental cost-effectiveness ratio (ICER) at or below $3,781. When we considered biochemical verification, sensitivity analysis, and subgroups, the costs per quit were less than $2,050 or the ICERs were less than $6,800.

**Conclusion:**

All community-based interventions included in this review were cost-effective. When economic evaluation results are extrapolated to future savings, the low cost per quit or ICER indicates that the cost-effectiveness of community-based tobacco dependence treatments is similar to the cost-effectiveness of clinic-based programs and that community-based interventions are a valuable approach to tobacco control. Additional research that more fully characterizes the cost-effectiveness of community-based tobacco dependence treatments is needed to inform future decisions in tobacco control policy.

**Summary**

**What is already known on this topic?**

Tobacco use poses a substantial economic burden on society, yet community-based interventions continue to be overlooked. Studies have shown tobacco dependence treatments, ranging from brief clinician advice to specialist-delivered intensive programs, are highly cost-effective.

**What is added by this report?**

This review addresses a gap in the scientific literature and demonstrates that community-based tobacco dependence treatments are cost-effective.

**What are the implications for public health practice?**

The low costs per quit and low incremental cost-effectiveness ratios of community-based programs indicate that these programs are a valuable component of comprehensive tobacco control. Efforts should continue to promote such programs.

## Introduction

In the United States, tobacco use, the leading cause of preventable morbidity and mortality (1), poses a substantial economic burden. Nationwide, smoking causes an estimated $332 billion in annual lost productivity and direct health care costs (1,2). Elimination of combustible tobacco use will dramatically reduce this burden (1).

Community-based cessation approaches, such as quitlines, internet, and self-help interventions, are important components of comprehensive tobacco control. Although community-based initiatives may have lower cessation rates (3) than clinic-based programs, they are effective as they engage more participants than do clinic-based programs, including underserved and hard-to-reach smokers (4–6). Because community-based interventions are often less expensive to deliver, they may be as cost-effective as clinic-based interventions. Furthermore, community-based programs offer an opportunity for tailored behavioral interventions to facilitate optimal outcomes and reduce tobacco use disparities (7).

Cessation interventions can immediately affect the economic and public health consequences of tobacco use (8). Yet comprehensive tobacco control efforts, which are typically organized at the state level and include administrative, surveillance, evaluation, and monitoring components, incur high implementation costs. These costs are allocated toward changing social norms, increasing cessation, and reducing exposure to secondhand smoke. It is essential that decision makers have tools such as economic evaluation to make informed decisions about how to allocate resources.

Few economic evaluations of controlled community-based trials in the United States have been published, and no comprehensive literature review exists. This void limits evidence-based decision making and resource allocation and negatively affects health outcomes. This review addresses the void by systematically assessing the cost-effectiveness and quality of economic evaluations of community-based tobacco dependence treatments conducted as randomized controlled trials in the United States. This report potentially informs tobacco control policy, contributes to population-level strategies, and improves future economic evaluations by illustrating best and current practices.

## Methods

We searched 8 databases (MEDLINE/PubMed, Tufts Cost-Effectiveness Analysis Registry, EMBASE, CINHAL, Scopus, Web of Science, Global Health, and the NHS Economic Evaluation Database) by using medical subject headings and synonyms for tobacco dependence treatment, economic evaluation, and randomized controlled trial. We searched databases from their beginning to February 2018.

### Study selection

Selection criteria were structured by population, intervention, comparator, outcome, and study design (Box). We included economic evaluations that randomized individuals or communities to intervention or control groups. We excluded review articles but examined them for studies that met criteria. All outcome time points, perspectives (ie, societal, payer/provider/program, or individual), and types of economic evaluations were included. We also included studies without biochemical verification. Our review focused on community-based tobacco dependence treatments, such as those delivered through quitlines/telephone counseling, in-person counseling, postal mail, and the internet, and included alternatives to traditional, medically trained clinical interventions. Although “community-based” has several definitions, we defined it as programs that 1) referred to a community as a setting or a geographic location where interventions were implemented, 2) included a variety of approaches, levels, and locations, and 3) focused on changing individuals’ behavior as a method of reducing population risk (9). Locations included state quitlines and local and population-based residents throughout the United States (reached by postal mail, internet, telephone counseling, and in-person counseling). Only studies with adult smokers and abstinence-framed measures such as cost per quit and incremental cost-effectiveness ratio (ICER) (ie, attributable cost per quit) were included. We excluded special or targeted populations such as adolescents, employees, insured, and hospitalized patients; studies conducted outside of the United States; and studies solely reporting outcomes in terms of enrollees, patients, or recruitment.

**Box. Selection Criteria Structured by Population, Intervention, Comparator, Outcome, and Study Design (PICOS)**

**Table:** 

Element	Inclusion Criteria	Exclusion Criteria
Population	Adult smokers; United States; community-based (accessible to broader populations, including socioeconomically disadvantaged populations)	Adolescents, worksite/employees, clinics, hospitals/inpatients, pregnant women, former smokers, insured, groups with specific conditions (eg, cancer patients, substance abuse), non-US setting
Intervention	Tobacco dependence treatment; smoking cessation (includes quitline)	—
Comparator	Controls, usual care (includes quitline)	—
Outcome	Abstinence-framed outcomes, such as but not limited to cost per quit, incremental cost-effectiveness ratio, quality-adjusted life year	Outcomes framed only as patient, enrollee, or recruitment
Study design	Controlled trials with economic evaluation, which randomized individuals or communities to an intervention or control condition	Observational studies, studies that did not randomize individuals or communities to an intervention or control condition, studies without economic evaluation

The search strategy was reviewed by an academic research librarian. To avoid publication bias, we performed gray literature searches of ClinicalTrials.gov, conference reports and proceedings (The Conference Board and Conference Proceedings Citation Index), and dissertations (WorldCatDissertations and Theses) to include reports, book chapters, conference abstracts, and dissertation theses. To avoid language bias, we included all languages. All publications indexed were included without date restrictions.

We removed duplicates, and 2 researchers (S.A.R., S.K.) independently performed an initial screening of titles and abstracts. Publications remaining were reviewed in entirety to determine eligibility. References in included studies were reviewed. We contacted authors for additional information when necessary. The same 2 researchers independently abstracted data and assessed quality. Any disagreement or uncertainty was resolved through discussion or a third independent researcher (M.E.W.).

Initial searches returned 3,480 articles. After removing 993 duplicates, we screened titles and abstracts of 2,487 records. Of these, 2,419 were excluded, leaving 68 full-text articles that were systematically examined for the following criteria in the following order: presence of an economic evaluation, randomized controlled trial, community-based, and set in the United States. Of these 68 studies, 9 that were published in peer-reviewed journals met inclusion criteria (Figure).

**Figure:**
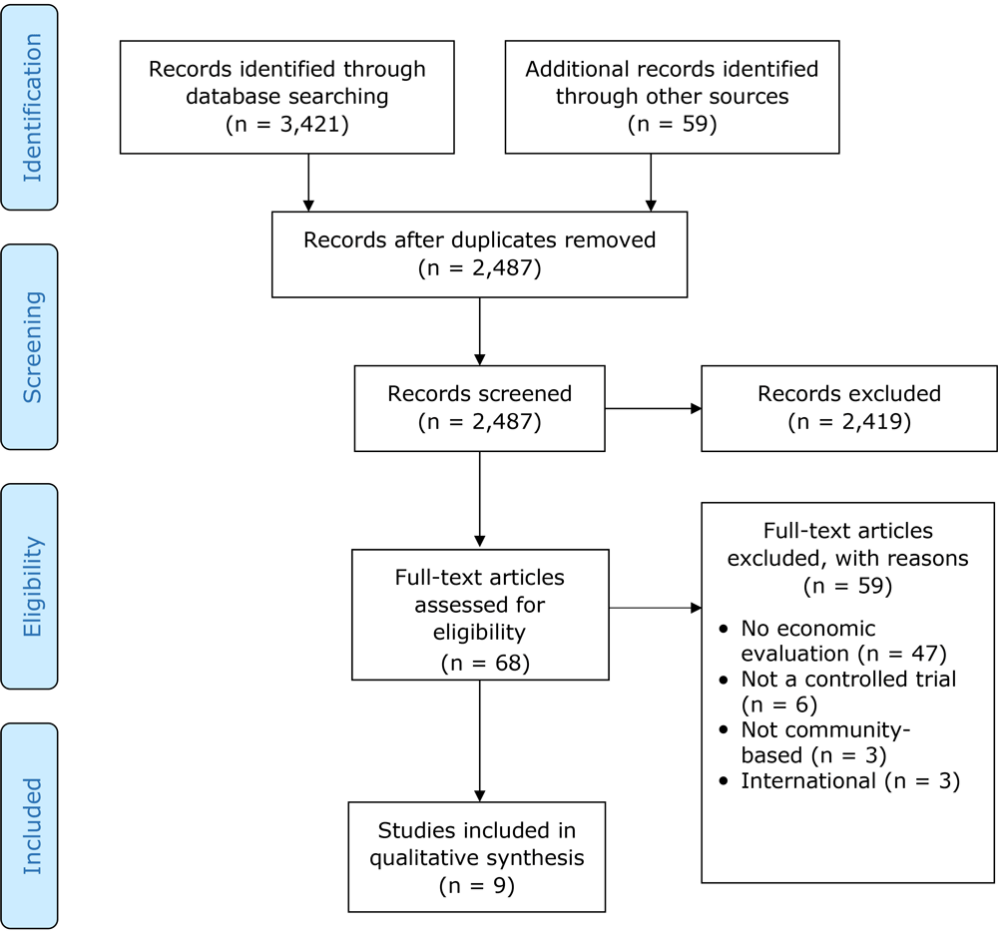
Article search and selection process using PRISMA (Preferred Reporting Items for Systematic Reviews and Meta-Analyses). “Records screened” are titles and abstracts.

### Data extraction

Two researchers (S.A.R., S.K.) independently abstracted data for 1) research question, objective, or hypothesis, 2) location or setting, 3) population description, 4) study design and intervention description, 5) intention-to-treat analyses, 6) summary effects, 7) type of economic evaluation, 8) perspective, 9) the analytic horizon, or the duration of the study time frame including outcome assessment (22), 10) type of abstinence measure used for the economic evaluation, 11) source of the valuation of resources (eg, invoices, contracts, website data) used to determine costs, or how components of intervention were valued, 12) sensitivity analyses, 13) economic evaluation results, 14) subgroup analyses, 15) generalizability, and 16) limitations. We assessed the quality of each study by using Drummond and Jefferson’s economic evaluation checklist (10), which consists of 35 questions that use 4 response options (yes, no, not clear, and not appropriate) to assess study design, sources and quality of data collected, data analysis, and interpretation of results. We noted any items not explicitly addressed but inferred from the text. We synthesized a qualitative narrative of outcomes. We assessed inter-rater reliability by calculating percentage agreement, expected agreement, and the Cohen κ statistic in Stata version 12 (StataCorp LLC). Cohen κ, values for which range from −1 to +1, represents the proportion of agreement after chance is excluded; larger values indicate better reliability. The statistic accounts for the possibility of guessing, but the assumptions of rater independence and other factors may lower the estimate of agreement, and interpretation and accepted values can vary by discipline (11,12).

## Results

For the initial review of titles and abstracts, the agreement rate was 98.6%, the expected agreement was 94.3%, and Cohen κ was 0.75. The agreement for full article review was 91.2%, the expected agreement was 72.7%, and Cohen κ was 0.68. The agreement for reasons of exclusion was 92.6%, the expected agreement was 76.3%, and Cohen κ was 0.69. The agreement for quality assessment was 93.9%, the expected agreement was 31.0%, and Cohen κ was 0.91.

**Study setting and implementation.** The primary setting for the 9 studies was quitline or telephone counseling (13–18). Other settings included in-person counseling (19), postal mail (17,20,21), and internet (14). Quitlines and telephone counseling were located in New York (18), Wisconsin (15), Oregon (16), Colorado (13), National Jewish Health (Denver, Colorado) (14), and the American Cancer Society in Texas (17). Studies were initiated over a wide time frame (1979–2010); 8 studies began in 2000 or later. Publication dates ranged from 1984 to 2016.

**Interventions.** Interventions tested the effect of type (15,18) and/or duration (13,15,16,19) of nicotine replacement therapy (NRT), duration and intensity of mailed self-help (20,21), quitline and self-help (17), and basic internet, enhanced internet, and enhanced internet plus telephone counseling (14) (Table 1).

**Table 1:** Interventions Described in 9 Studies Included in a Systematic Review of Studies Evaluating the Cost-Effectiveness of Tobacco Dependence Treatment Programs Delivered in Community-Based Settings

Study	Treatment Approach	Location or Setting, Study Period, Sample Size	Research Question, Objective, Hypothesis	Population	Effects/Outcomes
Krupski et al (18), 2016	Type of NRT used, quitline/telephone counseling	New York State; March 2010–October 2010 (N = 3,118)	Tested the provision of 2 weeks of either combination therapy (nicotine patch and nicotine lozenge) or monotherapy (nicotine patch alone) to heavy smokers calling the quitline.	Adult residents of New York State (≥18 y); self-identified as current daily tobacco user; ≥20 CPD; contacted the New York State Smokers’ Quitline; interested in using NRT; no known contraindications for NRT; and scored either 5 or 6 on the heaviness of smoking index (range 0–6).	Responder quit rates according to the protocol were higher for those given combination therapy than for those given monotherapy for 7-day (24% vs 21%) and 30-day (21% vs 18%) point prevalence abstinence, although they were not significantly different (*P* > .05). Results did not differ between intention-to-treat and according-to-protocol analyses.
Smith et al (15), 2013	Type of NRT used and duration of use, quitline/telephone counseling	Wisconsin; April 1, 2010–June 15, 2010 (N = 987)	Tested combination NRT (vs nicotine patch only), longer duration of NRT (6 vs 2 weeks), and medication adherence intervention (vs standard counseling).	Quitline callers aged ≥18 y; English speaking; ≥10 CPD; willing to set a quit date within next 30 days. Excluded: pregnant or lactating women; contraindications or unwillingness to use study medications.	Abstinence for combination NRT was higher (49.9%) vs nicotine patch only (42.3%); OR = 1.36 (95% CI, 1.06–1.75). No significant difference for 6 weeks vs 2 weeks; No difference in medication adherence counseling vs no medication adherence counseling; 30-day point prevalence abstinence was lower than 7-day point abstinence.
McAfee et al (16), 2008	Duration of NRT use, quitline/telephone counseling	Oregon Free Patch Initiative; October 18, 2004–May 5, 2005 (N = 1,154)	Evaluated the relative benefit and cost-effectiveness of short vs standard NRT treatment.	Uninsured quitline callers; aged ≥18 y; English speaking; had a working telephone; ≥5 CPD; no known contraindications for NRT; interested in using NRT and quitting in 30 days.	Intent-to-treat 30-day abstinence was 14.3% in the 2-week group and 19.6% in the 8-week group (OR 1.45; 95% CI, 1.01–2.12).
Burns et al (13), 2016	Duration of NRT use, quitline/telephone counseling	Colorado; March 2010–February 2011 (N = 1,495^a^)	Hypothesized that group receiving the smaller supply (4-week vs 8-week) NRT would have lower levels of abstinence. Secondary analyses of costs per quit, NRT utilization, and participant purchase of additional NRT.	Quitline callers; English speaking; 16–20 CPD; eligible for and willing to receive free patches; absence of a condition requiring physician approval for NRT.	Abstinence rates did not differ significantly between study conditions: 13.8% vs 12.4% in 4-week vs 8-week arms, respectively (30-day point-prevalence abstinence). NRT duration was similar in both groups, due in part to purchase of additional patches in 4-week group; About one-third of the 8-week group requested the full 8-week supply and had higher abstinence rates.
Schnoll et al (19), 2016	Duration of NRT use, in-person	University of Pennsylvania (academic center); October 2004–March 2008 (N = 568^b^)	Assessed the efficacy of extended (24 weeks) vs standard (8 weeks) transdermal nicotine therapy for promoting biochemically confirmed point prevalence abstinence at weeks 24 and 52 among adult smokers.	Adult treatment-seeking smokers recruited through advertisements for a free smoking cessation program; aged 18–65; ≥10 CPD for at least past year. Excluded: pregnancy or lactation, uncontrolled hypertension, unstable angina; heart attack or stroke within previous 6 months, recent diagnosis of cancer or kidney/liver failure, a history of organ transplant, current diabetes, drug or alcohol dependence, history of an Axis I psychiatric disorder, current use of a concomitant medication, or current treatment of nicotine addiction.	Odds of point prevalence abstinence were ~2 times greater for extended vs standard therapy at week 24 (31.6% vs 20.3%; OR = 1.81 [95% CI, 1.23–2.66]; *P* = .002). No difference at week 52.
McAlister et al (17), 2004	Quitline/telephone counseling	Texas; June 26, 2000–November 15, 2000 (N = 1,014)	Summarized 1-year follow-up results and cost-effectiveness estimates from a randomized trial designed to evaluate a new telephone counseling service established by the American Cancer Society in the summer of 2000.	Smokers agreeing to make a quit attempt within 2 weeks.	Maintained cessation rate was 10.3% in the group offered counseling and 5.8% in the group receiving booklets only. Net increment was 4.5% (χ^2^ test, *P* < .01)
Graham et al (14), 2013	Internet, quitline/telephone counseling	Throughout the United States; 2005–2007 (N = 2,005)	Conducted an economic evaluation of The iQUITT study, a randomized trial comparing basic internet, enhanced internet, and enhanced internet + phone counseling at 3, 6, 12, and 18 months.	Smokers recruited through active user interception sampling (entered terms “quit(ting) smoking,” “stop(ping) smoking,” or “smoking” in a major internet search engine and clicked on a link to www.quitnet.com); US residence; ≥5 CPD; ≥18 y; no prior use of QuitNet.	30-day point prevalence abstinence rates increased over time. Significant between-group differences in point prevalence were observed at 3, 6, and 12 months, but not at 18 months. Post hoc comparisons showed enhanced internet + phone outperforming the other 2 conditions at 3 and 6 months, and enhanced internet at 12 months (*P* < .003). The difference between enhanced internet and basic internet for point prevalence abstinence was not significant at any follow-up. 30-Day multiple point prevalence abstinence rates declined across groups over time. Significant between-group differences in multiple point prevalence were observed at all follow-ups with enhanced internet + phone outperforming other conditions. Difference between enhanced internet and basic internet for multiple point prevalence abstinence was not significant at any time.
Brandon et al (20), 2016	Self-help, postal mail	Throughout United States; April 2010–August 2011 (N = 1,874)	Hypothesized extended self-help would be more effective than traditional self-help and that increasing the intensity and duration of the intervention would produce enhanced efficacy in a dose–response manner. Extended self-help would continue to produce favorable cost-effectiveness compared with traditional smoking-cessation interventions.	National sample of daily smokers, recruited nationally via multimedia advertisements; aged >18 y; smoked ≥5 CPD during past year; English speaking and reading; desire to quit smoking, indicated by a score of 5 (“Think I should quit, but not quite ready”) or higher on the Contemplation Ladder (a measure of readiness to consider smoking cessation); not currently enrolled in a face-to-face smoking-cessation program.	A dose–response effect was found across all 4 follow-up points. By 24 months, intensive repeated mailings produced the highest abstinence rate (30.0%), followed by standard repeated mailings (24.4%), and traditional self-help (18.9%). Difference in 24-month abstinence rates between intensive repeated mailings and traditional self-help was 11.0% (95% CI, 5.7%–16.3%).
Davis et al (21), 1984	Self-help, postal mail	5 Local lung associations: San Diego, California; Salinas, California; Minneapolis/St. Paul, Minnesota; Baltimore, Maryland; and New York, New York; 1979–1981 (N = 1,237)	Examined long-term results of self-help smoking cessation programs involving no face-to-face contact during treatment.	Smokers responding to lung association announcements using standard newspaper advertisements provided by American Lung Associate, flyers, and media announcements.	20% quit initially, with 5% continually abstinent in cessation manual + maintenance manual at 12 months vs 2% for leaflets (*P* < .05). Nonsmoking prevalence rates gradually increased after 6 months. At 12 months, those with a maintenance component, (leaflet + maintenance manual and cessation manual + maintenance manual) had higher rates (18%) than leaflet alone (12%) or cessation manual alone (15%).

Abbreviations: CI, confidence interval; CPD, cigarettes per day; NRT, nicotine replacement therapy; OR, odds ratio.

a 1,503 study participants were enrolled, but 8 were never sent NRT, resulting in 1,495 study participants.

b A priori sample size was 600; 575 were randomized, but 7 people were ineligible because of medical contraindications after randomization and excluded from intention-to-treat analyses.

**Study populations.** Study populations varied; generally, participants were English-speaking, middle aged, white, motivated to quit, and highly nicotine dependent. Key factors that differed were number of cigarettes consumed or level of nicotine dependence, smoking history, interest or willingness to receive or use NRT, willingness to quit smoking, and sociodemographic characteristics.

**Measures of abstinence.** All studies provided details about how treatment effectiveness was defined and measured. The most commonly used definitions of abstinence for the economic evaluation were self-reported 7-day point prevalence (15,18–20) and 30-day point prevalence (13,14,16,21). Other definitions included multiple point prevalence (14), continuous abstinence (21), and maintained cessation with no more than 5 single-day slips (brief relapses or resumption of smoking for the delineated period of time) in a 3-month interval (17). The most common time points were 6 months (13–16,19) and 12 months (14,17,21). Other time points included 3 months (14), 7 months (18), 18 months (14), and 24 months (20). All studies addressed intention-to-treat analysis, in which nonrespondents were classified as smokers. However, 2 studies used intention-to-treat analyses in slightly different ways (16,18). Most studies defined abstinence on self-reported data (13–16,18,20,21), but 2 used biochemical verification to varying degrees (17,19). Seven studies (13,18) demonstrated a significant treatment effect (ie, difference in abstinence).

**Perspective.** Three studies explicitly stated the perspective (14,16,20). Among the remaining studies, perspective could be inferred (13,15,17–19,21). All studies used the payer/provider/program perspective; 1 study examined costs incurred by participants (ie, an individual perspective), but this approach was excluded in the original study’s final analyses (19).

**Costs.** Overall, studies provided various levels of detail on costs (Table 2). Four studies indicated the resource that was used to determine how the component was valued (ie, source of the valuation) (13,14,19,21). When reported, information on costs was typically obtained from prevailing commercial costs, economies of scale, or study records. Most studies reported aggregate data with various levels of detail about which intervention components were included.

**Table 2:** Description of Costs in 9 Studies Included in a Systematic Review of Studies Evaluating the Cost-Effectiveness of Tobacco Dependence Treatment Programs Delivered in Community-Based Settings

Study	Costs per Person	Source of Valuation^a^ and Comments
Krupski et al (18), 2016	Patches: $21 for 2 weeks	No source of valuation was provided.
	Patches + lozenges: $87 for 2 weeks (14 patches + 144 lozenges)	
Smith et al (15), 2013	Patches: $178 for 2 weeks	No source of valuation was provided. Costs included direct costs associated with registration, provision of NRT and counseling, and mailing of a quit guide (all participants), facility space, supplies, and physician supervision time.
	Patches: $233 for 6 weeks	
	Patches + gum: $213 for 2 weeks	
	Patches + gum: $348 for 6 weeks	
McAfee et al (16), 2008	Counseling + patches: $165.82 for 2 weeks	No source of valuation was provided. Costs included telephone counseling, mailed self-help quit kit, and NRT.
	Counseling + patches: $275.40 for 8 weeks	
Burns et al (13), 2016	Patches: $54 for 4 weeks	Source of valuation: Colorado Department of Public Health and Environment.
	Counseling/calls: $37.50 for the first call	
	Counseling/calls: $28.55 for each subsequent call	
Schnoll et al (19), 2016	Patches: $140 for 8 weeks	Source of valuation for NRT: www.drugstore.com. No source of valuation was provided for counseling. Costs included both direct and indirect costs.
	Patches: $420 for 24 weeks	
	Counseling/calls: $120 for both arms of the intervention (in-person)	
McAlister et al (17), 2004	Counseling/calls: $60	No source of valuation was provided. Cost estimates include staffing, fulfillment, telephone, evaluation, overhead, and infrastructure costs. Cost of taking calls and mailing self-help books to smokers who want to quit, which was the current practice at the call center, was approximately $15 for each smoker served.
Graham et al (14), 2013	Basic internet: $1	Estimated real-world commercial cost at scale for static web page.
	Enhanced internet: $40	QuitNet premium service for enhanced internet actual cost.
	Enhanced internet + phone: $145	Enhanced + phone includes at-scale charges.
Brandon et al (20), 2016	Traditional self-help: $5.46	No source of valuation was provided. Costs include printing, postage, and handling costs per intervention condition.
	Standard repeated mailings: $36.12	
	Intensive repeated mailings: $45.50	
Davis et al (21), 1984	Costs for recruitment, training of interviewers, and the telephone follow-up interviews. Costs were evenly allocated among all 4 experimental groups (leaflets; leaflets + maintenance manual; cessation manual; cessation + maintenance manuals). Costs: $12,451 recruitment; $4918.50 interview, assuming $4.50 hourly wage and 1,093 hours, utilities not included. Total direct costs for staff time were $1,700 with training costs prorated for 11 days using an assumed $20,000 annual salary.	Costs for the printing (not development) of the self-help materials, handling, and postage costs at the time of the study (1979–1981), which were prorated to the number of participants in each group, but data/values were not provided. Costs of recruitment, available from 4 of the test sites, were statistically imputed for the fifth site (in Salinas, California) by prorating the average from the other 4 sites by the number of participants recruited at that site.

a Resources (eg, invoices, contracts, website data) used to determine costs, or how components of intervention were valued.

**Analytic horizon.** The analytic horizon was explicitly addressed by 1 study (14) and easily understood in all other studies. Horizons varied from 3 to 24 months; most were 6 or 7 months (13–16,18), and one was a single 24-month follow-up study (20). One study had multiple periods of assessment (3, 6, 12, and 18 months) (14).

**Discounting.** Discounting was not performed in any of the studies but was discussed in one (14).

**Sensitivity analyses.** Many studies provided sufficient details of statistical tests and confidence intervals (13–20). Some degree of sensitivity analysis or uncertainty analysis was performed in relation to the economic evaluation in 5 studies (14,16,18,19,21); no study varied costs. Two studies used 95% confidence intervals (18,19), two used varied abstinence measures (14,21), and one examined the effect of using responder-only data in lieu of intention-to-treat data (16).

**Cost-effectiveness.** All studies conducted cost-effectiveness analyses, used cost per quit and/or ICER to present findings, and answered the study question posed. The combination of cost per quit and ICER was commonly used (14–16,18). Two studies calculated cost per quit only (13,21) and 3 studies reported ICER only (17,19,20). Studies compared relevant alternatives with various levels of detail (13–16,18–21). Data and results were typically presented in disaggregated and aggregated form to allow readers to calculate other ratios (14–21). Conclusions were provided for all but 1 study (17).

Despite various abstinence definitions, assessment time points, and scale of tobacco dependence treatments, the cost-effectiveness estimates were relatively similar (Table 3). Overall, when combining all studies, despite settings and methodology differences, cost per quit ranged from $5 (14) to $2,040 (16) and the ICER or cost per additional quit ranged from $357 (15) to $3,781 (14). When considering sensitivity analyses and the use of biochemically verified data, the ICER increased to $6,781 (19).

**Table 3:** Economic Evaluation Summary Data in 9 Studies Included in a Systematic Review of Studies Evaluating the Cost-Effectiveness of Tobacco Dependence Treatment Programs Delivered in Community Based Settings^a^

Study	Conditions	Abstinence Measure	Time Point	Cost per Quit	ICER	Sensitivity Analyses	Subgroup
Krupski et al (18), 2016	2 weeks monotherapy	7-day point prevalence	7 months	$102.44	—c	$667–$2276	Uninsured: ICER $647 ($296–$3,438)
	2 weeks combination therapy			$362.50	$1,886		
Smith (15), 2013	2 weeks patch only	7-day point prevalence	6 months	$464	—c	—d	—d
	6 weeks patch only			$505	$712		
	2 weeks combination NRT			$442	$357		
	6 weeks combination NRT			$675	$1,290		
McAfee et al (16), 2008	2 weeks patch	Complete abstinence from tobacco for ≥30 days	6 months	$1,658	—c	Intent-to-treat responders only: cost per quit was $564 for 2 weeks and $738 for 8 weeks. ICER was $1,384	—d
	8 weeks patch			$2,040	$3,131		
Burns et al (13), 2016^e^	4 weeks patch	30-day point prevalence	6 months	$883	—c	—d	Cost per quit among those who received 8 weeks of NRT: $1,010
	8 weeks patch			$1,148			
Schnoll et al (19), 2016	8 weeks patch	Biochemically verified (carbon monoxide ≤10 ppm) 7-day point prevalence	24 weeks	—b	—c	95% CI, $1,519–$6,781	—d
	24 weeks patch				$2,482		
McAlister et al (17), 2004	Mailed self-help booklets	Maintained cessation (≤5 single-day slips in a 3-month interval)	12 months	—b	—c	—d	—d
	Booklets, eligible for telephone counseling				$1,300		
Graham et al (14), 2013	Basic internet	30-day point prevalence	3 months	$11	—c	Multiple measures of cost per quit and ICER	Adherence cost per quit in enhanced internet + phone: $346 was optimal scenario. In enhanced internet, $164 was optimal scenario.
	Enhanced internet			$383	$4,227		
	Enhanced internet + phone			$765	$1,197		
	Basic internet	30-day point prevalence	6 months	$8	—c		
	Enhanced internet			$277	$2,305		
	Enhanced internet + phone			$736	$1,841		
	Basic internet	30-day point prevalence	12 months	$6	—c		
	Enhanced internet			$266	—c		
	Enhanced internet + phone			$675	$1,528		
	Basic internet	30-day point prevalence	18 months	$5	—c		
	Enhanced internet			$230	—c		
	Enhanced internet + phone			$741	$3,781		
	Basic internet	30-day multiple point prevalence	3 months	$11	—c		
	Enhanced internet			$383	$4,227		
	Enhanced internet + phone			$765	$1,197		
	Basic internet	30-day multiple point prevalence	6 months	$15	—c		
	Enhanced internet			$543	$8,453		
	Enhanced internet + phone			$1165	$1,995		
	Basic internet	30-day multiple point prevalence	12 months	$22	—c		
	Enhanced internet			$840	—c		
	Enhanced internet + phone			$1,529	$2,176		
	Basic internet	30-day multiple point prevalence	18 months	$28	—c		
	Enhanced internet			$898	$5,072		
	Enhanced internet + phone			$1,882	$3,123		
Brandon et al (20), 2016	Traditional self-help	7-day point prevalence	24 months	—b	—c	—d	—d
	Standard repeated mailings				$560		
	Intensive repeated mailings				$361		
Davis et al (21), 1984	Leaflets	30-day point prevalence	12 months	$135	—c	Varied abstinence measures	—d
	Leaflets + maintenance manual			$105			
	Cessation manual			$126			
	Cessation manual + maintenance manual			$116			
	Leaflets	Continuous	12 months	$921	—c		
	Leaflets + maintenance manual			$497			
	Cessation manual			$669			
	Cessation manual + maintenance manual			$396			

Abbreviations: ICER, incremental cost-effectiveness ratio; NRT, nicotine replacement therapy; ppm, parts per million.

a Treatment approach for each study is noted in Table 1.

b Measure not calculated; study reported only ICER.

c Reference case or analysis not reported.

d Analyses not conducted.

e Main study effects were not significant.

**Cost per quit.** When comparing combination therapy to monotherapy and duration of NRT therapy, using actual estimates only (excluding subgroup and sensitivity analyses), cost per quit ranged from $102.44 (18) (2 weeks of patch only) to $675 (15) (6 weeks of combination therapy). In studies examining duration of patch-only interventions, cost per quit ranged from $883 (4 weeks) (13) to $2,040 (8 weeks) (16). When examining dose or intensity of self-help interventions, cost per quit ranged from $5 for basic internet to $1,882 for enhanced internet plus phone (14). Conditions such as enhanced internet (14), mailed leaflets, leaflets plus maintenance manual, cessation manual, and cessation manual plus maintenance manual (21) were comparable.

**ICER.** Similar to cost per quit, when comparing combination therapy, monotherapy, and duration of NRT therapy, using actual estimates only, the ICER ranged from $357 (15) (2 weeks of combination therapy) to $3,131 (16) (8 weeks of patch only). When considering sensitivity analyses and the use of biochemically verified data, the range increased to $6,781 (19). When examining dose or intensity of self-help interventions, the ICER ranged from $361 (20) (intensive repeated mailings) to $3,781 (14) (enhanced internet plus phone). Compared with mailed self-help booklets alone, the cost per additional quit attributable to telephone counseling availability was approximately $1,300 (17).

**Subgroup analysis.** Three studies included subgroup analyses in the economic evaluation, including a comparison of combination therapy and monotherapy among uninsured participants (18), differences in abstinence by intervention use (14), and the effect of requesting 2 NRT shipments (13).

**Generalizability.** Two studies reported that the study design translated to real world or national samples (15,20). One study noted uncertainty about whether the sample would generalize to other smokers (14); others indicated the eligibility criteria (13,19) or intervention type (21) might limit the representativeness.

**Quality.** The quality of the economic evaluations varied; generally, studies addressed most recommended items (Table 4) (10). Either explicitly or implied, 1 study addressed all 35 items in Drummond and Jefferson’s economic evaluations checklist (14), 6 studies described 89% to 97% of applicable items (15,16,18–21), and 2 studies reported on 80% of applicable items (13,17). Many topics, such as research question, viewpoint, costing, currency, time horizon, discounting, and sensitivity analysis, were implied. Although Drummond and Jefferson’s recommendations do not specify reporting of financial support, 8 studies reported federal (14,15,18–20), state (13,16–18), institutional (20), or industry (13) support.

**Table 4:** Quality Assessment of 9 Studies Included in a Systematic Review of Studies Evaluating the Cost-Effectiveness of Tobacco Dependence Treatment Programs Delivered in Community Based Settings^a^

Item	Krupski et al (18), 2016	Graham et al (14), 2013	Schnoll et al (19), 2016	Smith (15), 2013	Brandon et al (20), 2016	McAfee et al (16), 2008	Burns et al (13), 2016	Davis et al (21), 1984	McAlister et al (17), 2004
**Study design**									
1. The research question is stated	Yes^b^	Yes	Yes^b^	Yes^b^	Yes	Yes^b^	Yes^b^	Yes^b^	Yes
2. The economic importance of the question is stated	Yes	Yes	Yes	Yes^b^	Yes	Yes	Yes	Yes^b^	Yes
3. The viewpoint(s) of the analysis are clearly stated and justified	Yes^b^	Yes	Yes^b^	Yes^b^	Yes	Yes	Yes^b^	Yes^b^	Yes^b^
4. The rationale for choosing the alternative programmes or interventions compared is stated	Yes	Yes	Yes	Yes	Yes	Yes	Yes	Yes	Yes
5. The alternatives being compared are clearly described	Yes	Yes	Yes	Yes	Yes	Yes	Yes	Yes	Yes
6. The form of economic evaluation used is stated	Yes	Yes	Yes	Yes	Yes	Yes	Yes	Yes	Yes
7. The choice of form of economic evaluation is justified in relation to the question addressed	Yes	Yes	Yes	Yes	Yes	Yes	Yes	Yes	Yes
**Data collection**									
8. The source(s) of effectiveness estimate used are stated	Yes	Yes	Yes	Yes	Yes	Yes	Yes	Yes	Yes
9. Details of the design and results of effectiveness study are given (if based on a single study)	Yes	Yes	Yes	Yes	Yes	Yes	Yes	Yes^b^	Yes
10. Details of the method of synthesis or meta-analysis of estimates are given (if based on an overview of a number of effectiveness studies)	NA	NA	NA	NA	NA	NA	NA	NA	NA
11. The primary outcome measures for the economic evaluation are clearly stated	Yes	Yes	Yes	Yes	Yes	Yes	Yes	Yes	Yes
12. Methods to value health states and other benefits are stated	NA	NA	NA	NA	NA	NA	NA	NA	NA
13. Details of the subject from whom valuations were obtained are given	NA	NA	NA	NA	NA	NA	NA	NA	NA
14. Productivity changes (if included) are reported separately	NA	NA	NA	NA	NA	NA	NA	NA	NA
15. The relevance of productivity changes to the study question is discussed	NA	NA	NA	NA	NA	NA	NA	NA	NA
16. Quantities of resources are reported separately from their unit costs	Yes	Yes	Yes^b^	Yes^b^	Yes^b^	Yes^b^	Not clear	Yes^b^	Yes
17. Methods for the estimation of quantities and unit costs are described	Yes^b^	Yes	Yes^b^	Yes^b^	Yes^b^	Yes^b^	Yes^b^	Yes^b^	Yes^b^
18. Currency and price are recorded	Yes^b^	Yes^b^	Yes^b^	Yes^b^	Yes^b^	Yes^b^	Yes^b^	Yes^b^	Yes^b^
19. Details of currency and price adjustments for inflation or currency conversion are given	Yes^b^	Yes^b^	Yes^b^	Yes^b^	Yes^b^	Yes^b^	Yes^b^	Yes^b^	Yes^b^
20. Details of any model used are given	NA	NA	NA	NA	NA	NA	NA	NA	NA
21. The choice of model used and the key parameters on which it is based are justified	NA	NA	NA	NA	NA	NA	NA	NA	NA
**Analysis and interpretation of results**									
22. Time horizon of costs and benefits is stated	Yes^b^	Yes	Yes^b^	Yes^b^	Yes^b^	Yes^b^	Yes^b^	Yes^b^	Yes^b^
23. The discount rate(s) is stated	Yes^b^	Yes^b^	Yes^b^	Yes^b^	Yes^b^	Yes^b^	Yes^b^	Yes^b^	Yes^b^
24. The choice of rate(s) is justified	Yes^b^	Yes	Yes^b^	Yes^b^	Yes^b^	Yes^b^	Yes^b^	Yes^b^	Yes^b^
25. An explanation is given if costs or benefits are not discounted	No	Yes	No	No	No	No	No	No	No
26. Details of statistical tests and confidence intervals are given for stochastic data	Yes	Yes	Yes	Yes	Yes	Yes	Yes	No^b^	Yes
27. The approach to sensitivity analysis is given	Yes	Yes^b^	Yes^b^	No	No^b^	Yes^b^	No	Yes^b^	No
28. The choice of variables for sensitivity analysis is justified	Yes^b^	Yes^b^	Yes	No	No	Yes^b^	No	Yes^b^	No
29. The ranges over which the variables are varied are stated	Yes	Yes	Yes	No	No	Yes	No	Yes^b^	No
30. Relevant alternatives are compared	Yes^b^	Yes	Yes	Yes^b^	Yes	Yes	Yes	Yes	No
31. Incremental analysis is reported	Yes	Yes	Yes	Yes	Yes	Yes	No	No^b^	Yes
32. Major outcomes are presented in a disaggregated as well as aggregated form	Yes	Yes	Yes	Yes	Yes	Yes	Not clear	Yes	Yes
33. The answer to the study question is given	Yes	Yes	Yes	Yes	Yes	Yes	Yes	Yes	Yes
34. Conclusions follow the data reported	Yes	Yes	Yes	Yes	Yes	Yes	Yes	Yes	No^b^
35. Conclusions are accompanied by the appropriate caveats	Yes	Yes	Yes	Yes	Yes	Yes	Yes	Yes	No
Number of items categorized as no or not clear	1	0	1	4	4	1	7	3	7
Percentage of items categorized as yes or yes inferred from text	97%	100%	97%	89%	89%	97%	80%	91%	80%

Abbreviation: NA, not appropriate.

a Drummond and Jefferson’s economic evaluation checklist (10) was used to assess quality.

b Inferred from text.

## Discussion

Our review systematically assessed the cost-effectiveness and quality of the economic evaluations of community-based tobacco dependence treatment interventions conducted as randomized controlled trials in the United States. The studies reviewed addressed most of Drummond and Jefferson’s recommendations for authors and peer reviewers of economic submissions; however, some studies provided limited details. Based on cost-effectiveness estimates reported among studies of predominantly middle-aged, white, motivated to quit, and highly nicotine dependent populations, basic internet had the lowest cost-effectiveness ratio. However, all interventions, even when considering biochemical verification, sensitivity analysis, and subgroup analysis, had a cost per quit of less than $2,050 or an ICER of less than $6,800. Thus, considering the most commonly accepted conservative threshold of $50,000 per quality-adjusted-life-year (QALY), all community-based interventions were cost-effective.

According to Drummond and Jefferson, cost-effectiveness comparisons among health care interventions should be made only when methods and settings are closely aligned (10). In our review, no studies used QALYs or a similar outcome, which would have allowed for direct comparison with studies on other health conditions. However, the cost-effectiveness estimates of community-based interventions in our review (ie, cost per quit or ICER at or below $2,040 or $3,781, respectively) were similar to estimates in studies of clinical settings (ie, a few hundred to a few thousand dollars per quit [23–29]). Although community-based tobacco dependence treatment cost-effectiveness estimates in our review align with previous clinical findings and findings of nonrandomized controlled studies, more research is needed before definitive comparisons can be made.

Treatment for tobacco use is considered the gold standard of health care cost-effectiveness (30). Ranging from brief clinician advice to specialist-delivered intensive programs, including NRT or other medications, such treatment has been shown to be highly cost-effective (23). Population-wide policy, systems, and environmental changes, such as increases in the unit price of tobacco products, comprehensive smoke-free policies, and media campaigns increase cessation rates by motivating users to quit, increasing demand for tobacco dependence treatment, and making it easier to quit (7,8,31–33). These approaches are most efficient and effective at reaching many people (7,31,33); however, policies and media campaigns require substantial resources, and like clinical interventions, they are often not tailored to specific populations in need, do little to directly address disparities in tobacco use and tobacco-attributable health outcomes, and fail to engage hard-to-reach populations, such as those with social, economic, or geographic constraints.

In contrast, community-based programs are frequently tailored to targeted audiences. Tailored programs and messages are more effective than standardized, nontailored interventions (34), and have a greater effect on health behavior because they are often perceived as more relevant than generic communication (35–37). These benefits in reach and efficacy, which occur largely through engagement in evidence-based community interventions, can help reduce disparities in tobacco use (7). Furthermore, community-based programs, such as those using community health workers, have strong potential for improving health outcomes (38). Although clinical and policy-based approaches are important, they are also labor intensive, costly, and do little to address subgroups of populations who are underserved. Community-based interventions are poised to focus on those gaps. This review shows community-based tobacco dependence treatment approaches are both effective and cost-effective and an important component of comprehensive tobacco control. With this additional information on the cost-effectiveness of community-based interventions, efforts should continue to promote such strategies.

Our study has several strengths. The systematic review used a comprehensive search strategy that was informed and reviewed by an academic research librarian. We used 2 independent researchers and a third to resolve conflicts with high interrater agreement. The search was performed in 8 databases and gray literature, which produced substantial overlap in returned results. The quality assessment used Drummond and Jefferson’s established criteria and checklist, which is recommended in Cochrane reviews to inform appraisal of the methodological quality of economic evaluations (39). The study was limited in scope to include economic evaluations of community-based studies of tobacco dependence interventions conducted as randomized controlled trials in the United States. We identified a considerable number of studies outside the United States, but differences across countries’ cost structures and health systems would have limited the validity of our review (10). Randomized controlled clinical trials were selected as the gold standard for estimating efficacy of treatment interventions and greatest internal validity.

Our study has several limitations. We excluded several target populations (ie, adolescents, employees, insured, and patients) and other settings (ie, clinical settings and worksites) to increase the reliability of the findings. The only groups included a priori were low-resource groups, such as the uninsured. Socioeconomically disadvantaged populations underutilize smoking cessation treatments and are studied infrequently. Although some groups (eg, mentally ill, people who use drugs) could potentially be classified as low-resource, we determined that the underlying conditions would further complicate generalization. A broad body of research on those groups could be reviewed separately.

Cost-effectiveness ranges in our review were generalities; the ranges were not adjusted, because we lacked dollar-year data. However, when we used presumed dollar-years, adjusted cost-effectiveness estimates for the oldest study (21) and the study with the highest reported cost per quit (16) were below $2,700 in 2019 dollars. These approximations demonstrate that our findings would retain low cost-effectiveness ratios if dollar-year data were available to adjust costs to a single referent year. We could not directly compare the cost-effectiveness estimates among the 9 studies in our review because of heterogeneous study components and the lack of a common base case among study interventions, which complicate the comparison of studies and the ability to draw definitive conclusions. The heterogeneity precluded meta-analysis; rather, we performed a narrative synthesis and quality assessment. This approach is consistent with previous studies, and in practice, economic evaluations addressing a particular question do not generally present sufficient detail to permit adjustments required for a meta-analysis (40). Additionally, critical appraisal of the quality and reporting of health economic studies also vary. Because no minimum methodologic criteria or scoring systems exist, our evaluation of study quality was subjective.

Our review demonstrates that although smoking is an important public health issue, few economic evaluations of community-based tobacco dependence treatment programs in the United States have been conducted. However, the cost-effectiveness estimates found in our review are a fraction of the generally accepted cost-effectiveness threshold of $50,000-per-QALY. Given the degree to which tobacco influences numerous health outcomes and productivity, when the results are extrapolated to future savings, the low cost per quit or ICER is evidence that community-based cessation interventions are of great value and good investments for health.

Community-based agencies face challenges as a result of lack of tobacco control funding. At the time of the Master Settlement Agreement (MSA), many states established tobacco control programs; however, in 2018 only $721.6 million (<3%) of the $27.5 billion collected from MSA payments and state tax revenue in the United States was spent on smoking cessation and adolescent prevention (41). The median CDC-recommended level of state funding in 2014 was $48 million (7). In 2018, only 1 state (Alaska) funded its tobacco control programs at the CDC-recommended level (41). The funding for state tobacco prevention and cessation in 2019 ranges from 2% to 98.1%, with a median of 19.3% (41). The American Lung Association reports that the median amount states invest in quitlines is $2.21 per smoker in the state (41). Funding for community-based programs is inadequate, and such limited support will continue to exacerbate smoking-related health care costs and disparities.

Our review revealed a lack of focus on economic evaluation of community-based tobacco dependence treatment programs in the United States. We demonstrated these programs are cost-effective. It is vital to expand this literature given the effect of tobacco on health and costs. To improve the state of the science and understanding of results, as well as identify potential opportunities for uptake of interventions, such studies should be conducted. Future studies should adhere to and explicitly address key components of Drummond and Jefferson’s economic evaluation guidelines. Adding consistent base cases and valuation, subgroup analyses, cost-utility analyses, and standardizing approaches, especially related to measurement (eg, abstinence and costs) will improve comparability between studies and expand use in policy decisions. Future tobacco control economic evaluation research should also examine populations and areas with a high prevalence of smoking (eg, low-resource groups), in-person counseling, and mobile interventions.
